# Biomimetic design and clinical application of Ti-6Al-4V lattice hemipelvis prosthesis for pelvic reconstruction

**DOI:** 10.1186/s13018-024-04672-5

**Published:** 2024-04-01

**Authors:** Zhuangzhuang Li, Yi Luo, Minxun Lu, Yitian Wang, Taojun Gong, Xuanhong He, Xin Hu, Jingjunjiao Long, Yong Zhou, Li Min, Chongqi Tu

**Affiliations:** 1 Department of Orthopedics and Orthopaedic Research Institute, West China Hospital, Sichuan University, Chengdu, Sichuan 610041 People’s Republic of China; 2 Model Worker and Craftsman Talent Innovation Workshop of Sichuan Province, No. 37 Guoxue Road, Chengdu, People’s Republic of China

**Keywords:** 3D-printed, Lattice structure, Hemipelvis prosthesis, Pelvic reconstruction, Tumor resection

## Abstract

**Objective:**

This study aims to biomimetic design a new 3D-printed lattice hemipelvis prosthesis and evaluate its clinical efficiency for pelvic reconstruction following tumor resection, focusing on feasibility, osseointegration, and patient outcomes.

**Methods:**

From May 2020 to October 2021, twelve patients with pelvic tumors underwent tumor resection and subsequently received 3D-printed lattice hemipelvis prostheses for pelvic reconstruction. The prosthesis was strategically incorporated with lattice structures and solid to optimize mechanical performance and osseointegration. The pore size and porosity were analyzed. Patient outcomes were assessed through a combination of clinical and radiological evaluations.

**Results:**

Multiple pore sizes were observed in irregular porous structures, with a wide distribution range (approximately 300–900 μm). The average follow-up of 34.7 months, ranging 26 from to 43 months. One patient with Ewing sarcoma died of pulmonary metastasis 33 months after surgery while others were alive at the last follow-up. Postoperative radiographs showed that the prosthesis’s position was consistent with the preoperative planning. T-SMART images showed that the host bone was in close and tight contact with the prosthesis with no gaps at the interface. The average MSTS score was 21 at the last follow-up, ranging from 18 to 24. There was no complication requiring revision surgery or removal of the 3D-printed hemipelvis prosthesis, such as infection, screw breakage, and prosthesis loosening.

**Conclusion:**

The newly designed 3D-printed lattice hemipelvis prosthesis created multiple pore sizes with a wide distribution range and resulted in good osteointegration and favorable limb function.

**Supplementary Information:**

The online version contains supplementary material available at 10.1186/s13018-024-04672-5.

## Introduction

The pelvis is a common anatomical site for primary malignant bone tumors as well as metastatic disease [1]. With the application of adjuvant chemotherapy and improvement in radiologic technology and surgical techniques, limb salvage surgery has become the standard treatment for pelvic tumors [2–4]. The utilization of metallic prostheses is the currently preferred reconstruction method following tumor resection. However, the complex anatomy of the pelvis as well as its load-bearing function pose a great challenge for surgeons. Until now, several prostheses have been developed for pelvic reconstruction, such as saddle prosthesis [5, 6], ice cream cone prosthesis [7, 8], and modular hemipelvis prosthesis [1, 9, 10]. However, when the ilium and acetabulum are completely removed, the saddle prosthesis and ice cream cone prosthesis cannot be fitted and fixed, which requires sufficient bone stock of the residual ilium for implant anchorage [11, 12]. In this situation, modular hemipelvis prosthesis has gained popularity for pelvic reconstruction due to its flexible features in surgery [13]. Nevertheless, implant integration with host bone is still unsatisfying, and the prosthesis relies on simple screw fixation. Therefore, the potential for complications in long-term follow-up remains high, such as screw breakage and prosthesis loosening [14].

In recent years, the development of 3D printing technology has enabled the fabrication of personalized prostheses and patient-specific instruments [15, 16]. Due to the unique advantage of design freedom for individual bone defects, 3D-printed hemipelvis prosthesis presents a compelling opportunity for pelvic reconstruction following tumor resection [2, 17–21]. Additionally, 3D printing allows for the creation of porous structures incorporated within the implant, which effectively reduces the elastic modulus and provides space for bone in-growth, thereby increasing osteointegration [22, 23]. Lattice structure refers to a 3D framework characterized by a network of interconnected struts or beams arranged in a repeating pattern. In medical devices and implants, lattice porous structures can provide lightweight, excellent interconnectivity, and good mechanical strength [24]. However, it is noted that the natural bone trabecular network is a complex and irregular porous structure [25]. Recent studies have shown that irregular lattice structures facilitate osteointegration due to the biomimetic property [26, 27]. However, regular lattice structure remains the currently used design in clinical prostheses. To our knowledge, there is no literature on the use of irregular lattice design in hemipelvis prostheses.

Therefore, this study aims to biomimetic design a new 3D-printed lattice hemipelvis prosthesis with irregular porous structures and evaluate its clinical efficiency for pelvic reconstruction following tumor resection, focusing on feasibility, osseointegration, and patient outcomes.

## Methods

### Patients

**Fig. 1:**
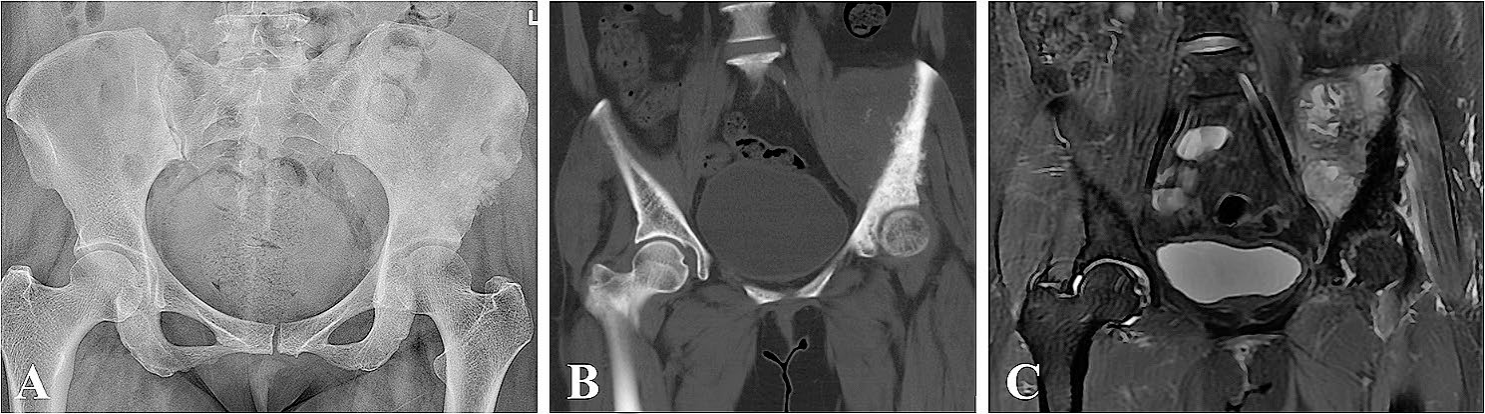
Preoperative X-ray (A), CT (B), and MRI (C) scans of a 50-year-old patient with osteosarcoma involving the acetabulum as well as most of the ilium

From January 2020 to October 2021, twelve patients with pelvic tumors underwent tumor resection and subsequently received 3D-printed lattice hemipelvis prostheses for reconstruction. Inclusion criteria were as follows: (1) pathological diagnosis of primary sarcoma, chondrosarcoma, or solitary metastatic lesion (2) lesions invade the acetabulum as well as most of the ilium, requiring complete removal of the ilium and acetabulum; (3) lesions do not extend to the sacroiliac joint; (4) follow-up period of more than 2 years. Exclusion criteria were: (1) life expectancy less than six months; (2) involvement of important neurovascular structures; (3) lack of complete follow-up information. Patient demographic and clinical characteristics, including gender, age, and pathological diagnosis were collected and shown in Table 1. Preoperatively, patients underwent detailed radiographic examinations of the pelvis, including X-ray, computed tomography (CT), and magnetic resonance imaging (MRI) (Fig. 1). The workflow for designing 3D-printed lattice hemipelvis implants was carried out after obtaining written informed consent from each patient.

**Table 1:** Demographics, clinical characteristics, and follow-up outcomes of twelve patients

Patients	Sex	Age, year	Dignosis	Resection type		Follow-up, months	MSTS score	Complications	Oncological status
1	F	36	Chondrosarcoma		I + II + III	43	21	-	NED
2	M	40	Chondrosarcoma		I + II + III	42	24	-	NED
3	M	66	Leiomyosarcoma		I + II + III	40	23	DWH	NED
4	M	35	Osteosarcoma		I + II	39	19	-	NED
5	F	65	Chondrosarcoma		I + II + III	37	20	DWH	NED
6	M	27	Ewing sarcoma		I + II + III	33	22	-	DOD
7	M	56	Lung cancer metastasis		I + II + III	36	22	-	AWD
8	F	66	Osteosarcoma		I + II + III	35	23	Dislocation	NED
9	M	36	Chondrosarcoma		I + II	30	21	-	NED
10	M	20	Osteosarcoma		I + II + III	28	18	-	NED
11	F	50	Osteosarcoma		I + II + III	27	22	-	NED
12	F	52	Chondrosarcoma		I + II + III	26	20	-	NED

DWH: delayed wound healing; NED: no evidence of disease; AWD: alive with disease; DOD: died of disease

### Personalized hemipelvis prosthesis design

**Fig. 2:**
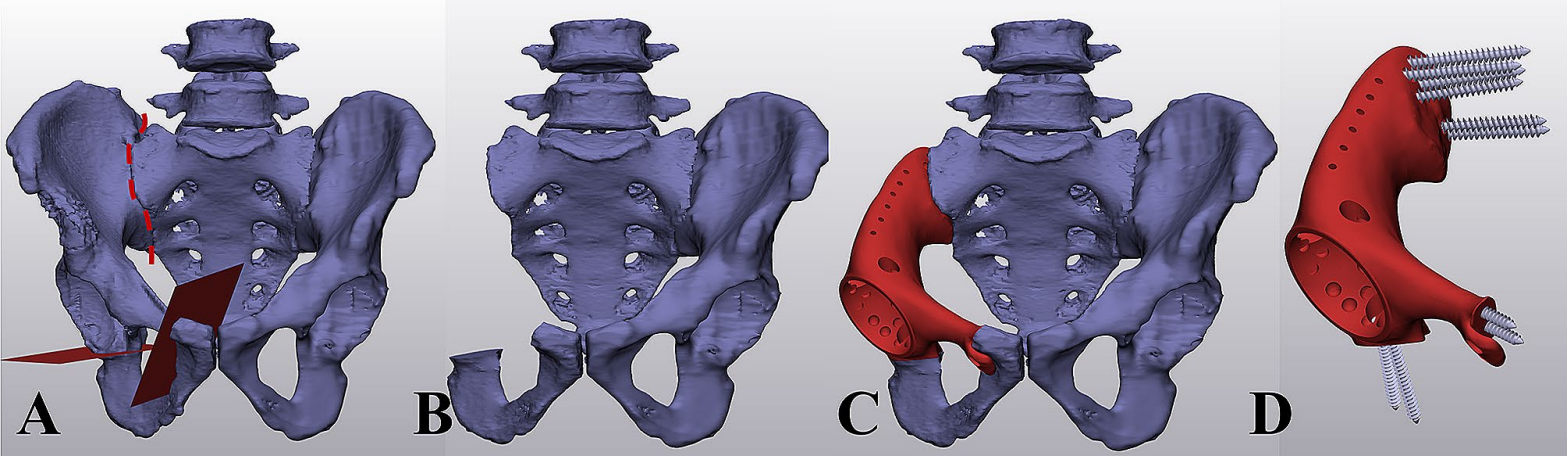
Profile of designing the personalized hemipelvis prosthesis for the individual bone defect. (A) determination of the osteotomy plane; (B) simulation of hemipelvis tumor resection based on the anatomical 3D model; (C, D) preliminary shape of the prosthesis with specific features, including plate, screw holes, and suture holes

The collected CT data (DICOM format) were imported into Mimics software (Materialise, Leuven, Belgium) to segment anatomical 3D models of the pelvis and tumor (Fig. 2). According to the MRI examination results, the tumor resection boundary was set as 10 mm for chondrosarcoma, 20 mm for metastasis lesions, and 30 mm for high-grade sarcomas. Following the determination of the resection location, the osteotomy plane of the pubic and ischial were set as perpendicular as possible to the axis of the bone. Then, hemipelvis tumor resection was simulated based on the anatomical 3D model. The preliminary shape of the prosthesis was designed by mirroring the 3D model of the contralateral hemipelvis. Then, the prosthesis shape was simplified, including minimizing the ilium wing and removing the posterior iliac spines. For all cases, the pubis superior ramus of the prosthesis model was preserved to reconstruct the pelvic ring. Then, specific features were added to the prosthesis model, including plate, screw holes, and suture holes.

**Fig. 3:**
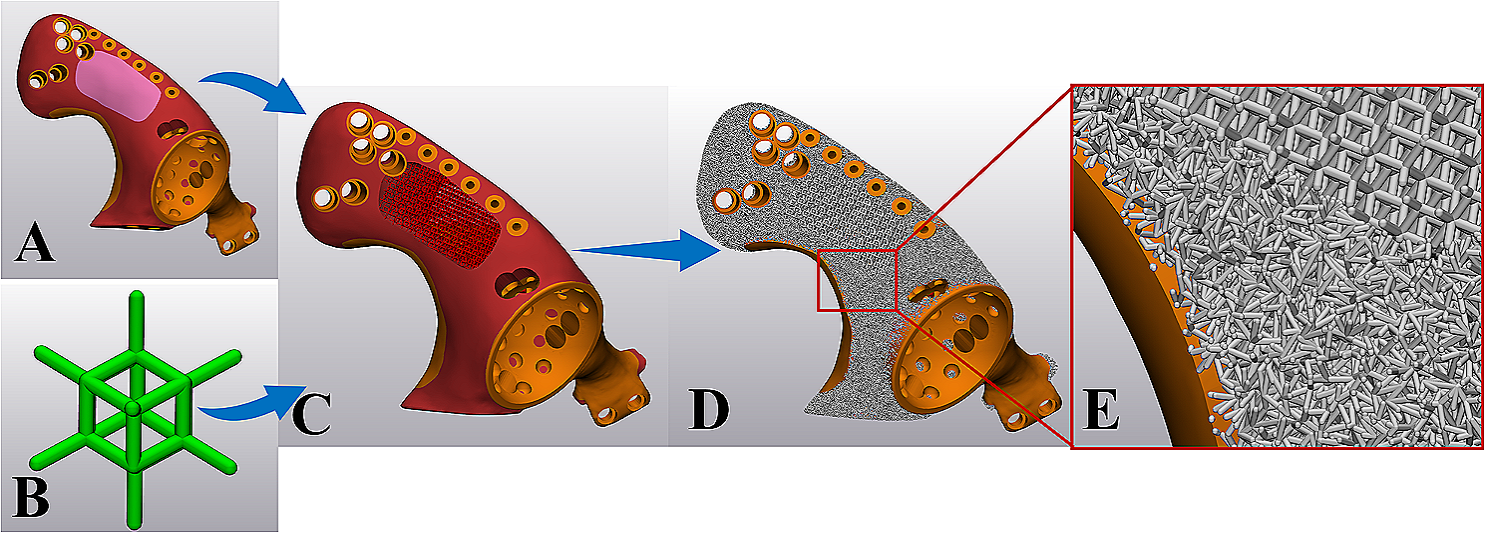
Profile of designing lattice structure for the hemipelvis prosthesis. (A) three parts were strategically incorporated to optimize mechanical performance and osseointegration; (B) dodecahedron cell was selected; (C) a regular large porous lattice part for reducing weight; (D, E) an irregular porous lattice part with the property to facilitate osteointegration

**Fig. 4:**
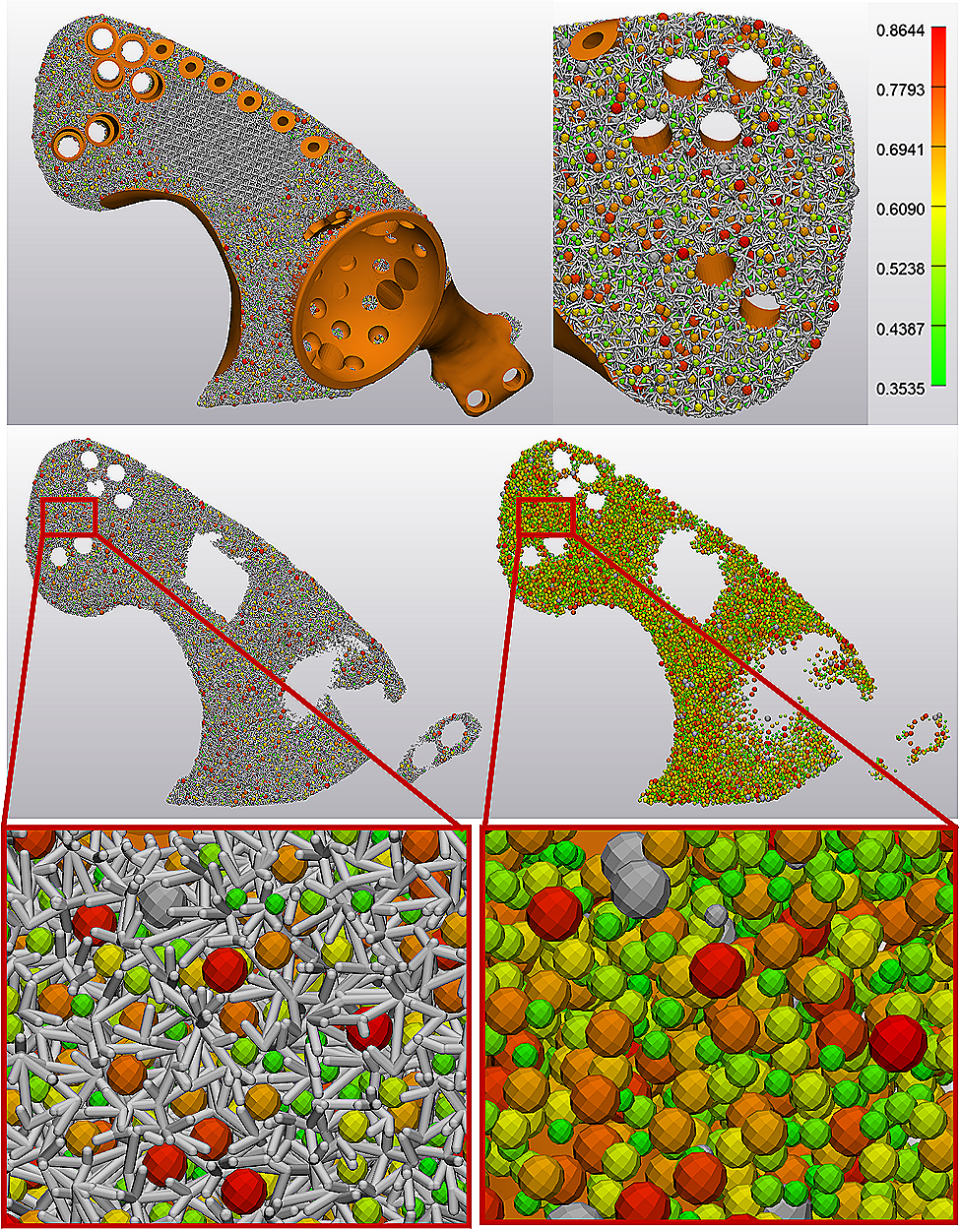
Pore size analysis of personalized lattice hemipelvis prosthesis for one patient. Multiple pore sizes were observed in the irregular porous lattice part with a wide distribution range (350–860 μm)

**Fig. 5:**
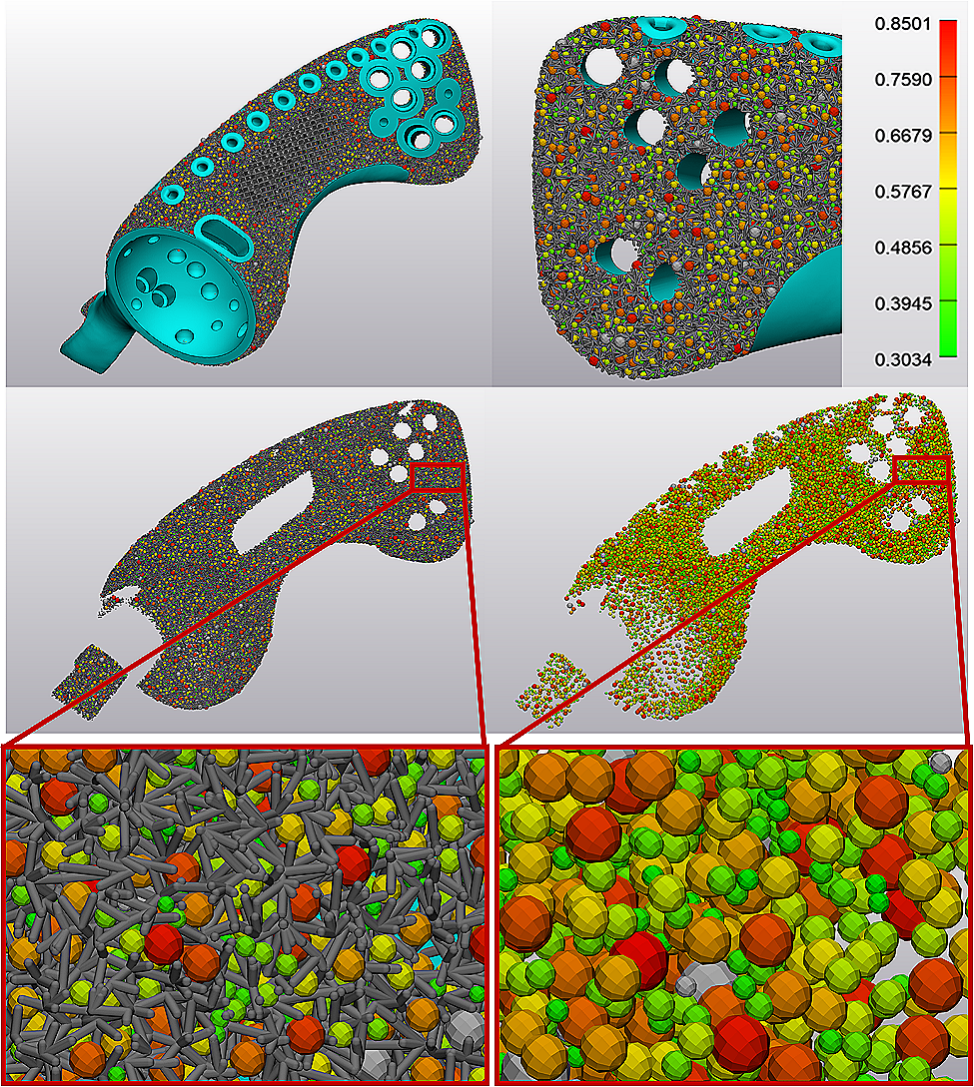
Pore size analysis of personalized lattice hemipelvis prosthesis for one patient. Multiple pore sizes were observed in the irregular porous lattice part with a wide distribution range (300–850 μm)

Hemipelvis prosthesis was strategically incorporated with lattice structures and solid to optimize mechanical performance and osseointegration (Fig. 3). 3-Matic (Materialise, Leuven, Belgium) software was employed to create the lattice structure, and the dodecahedron cell was selected. Unit cell parameters were adjusted for the irregular porous lattice part and regular large porous lattice part, respectively (Table 2). The pore size (Figs. 4 and 5) and porosity (Supplementary 1) were analyzed. The finalized prosthesis design file was exported in the STL format.

**Table 2:** Details of the personalized hemipelvis prosthesis and lattice structure design

Patients	Regular large porous parameters				Irregular porous parameters		Distribution of pore size in irregular part, um	Porosity in irregular part, %
	Unit cell size, mm	Unit cell strut thickness, um			Unit cell size, mm	Unit cell strut thickness, um		
1	5		600		2.6	400	350–860	75.2
2	5		600		2.6	400	330–870	74.9
3	5		600		2.6	400	340–860	75.6
4	5		600		2.6	400	310–860	74.7
5	5		600		2.6	400	310–850	75.1
6	5		600		2.6	400	320–860	75.2
7	5		600		2.6	400	350–860	75.1
8	5		600		2.6	400	320–840	74.9
9	5		600		2.6	400	310–850	75.0
10	5		600		2.6	400	330–870	74.8
11	5		600		2.6	400	300–870	75.1
12	5		600		2.6	400	310–880	74.7

### Ti-6Al-4V prosthesis manufacture

**Fig. 6:**
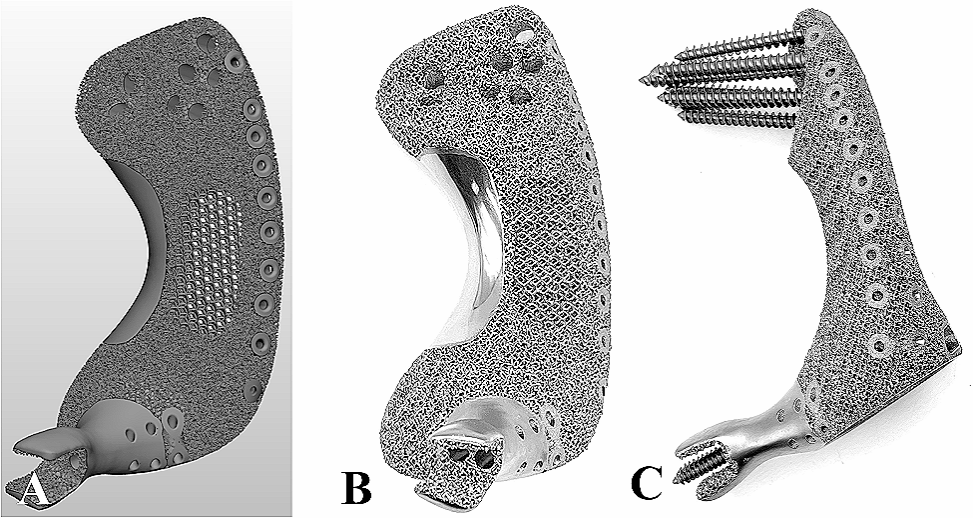
Final 3D model (A) and photos (B, C) of the lattice hemipelvis prosthesis for one patient

The lattice hemipelvis prostheses were manufactured using the electron beam melting (EBM) machine (ARCAM Q10plus, Mölndal, Sweden), utilizing the Ti-6Al-4V alloy powder. Then, the lattice hemipelvis prosthesis was polished, processed, and cleaned (Fig. 6). Prosthesis trial mold and patient-specific bone-cutting guides were fabricated by selective laser sintering with nylon powder. In addition, resin anatomical 3D models were prepared. Preoperatively, reconstruction of the pelvic defect with the 3D-printed lattice hemipelvis prosthesis was re-simulated was performed.

### Surgical procedure

**Fig. 7:**
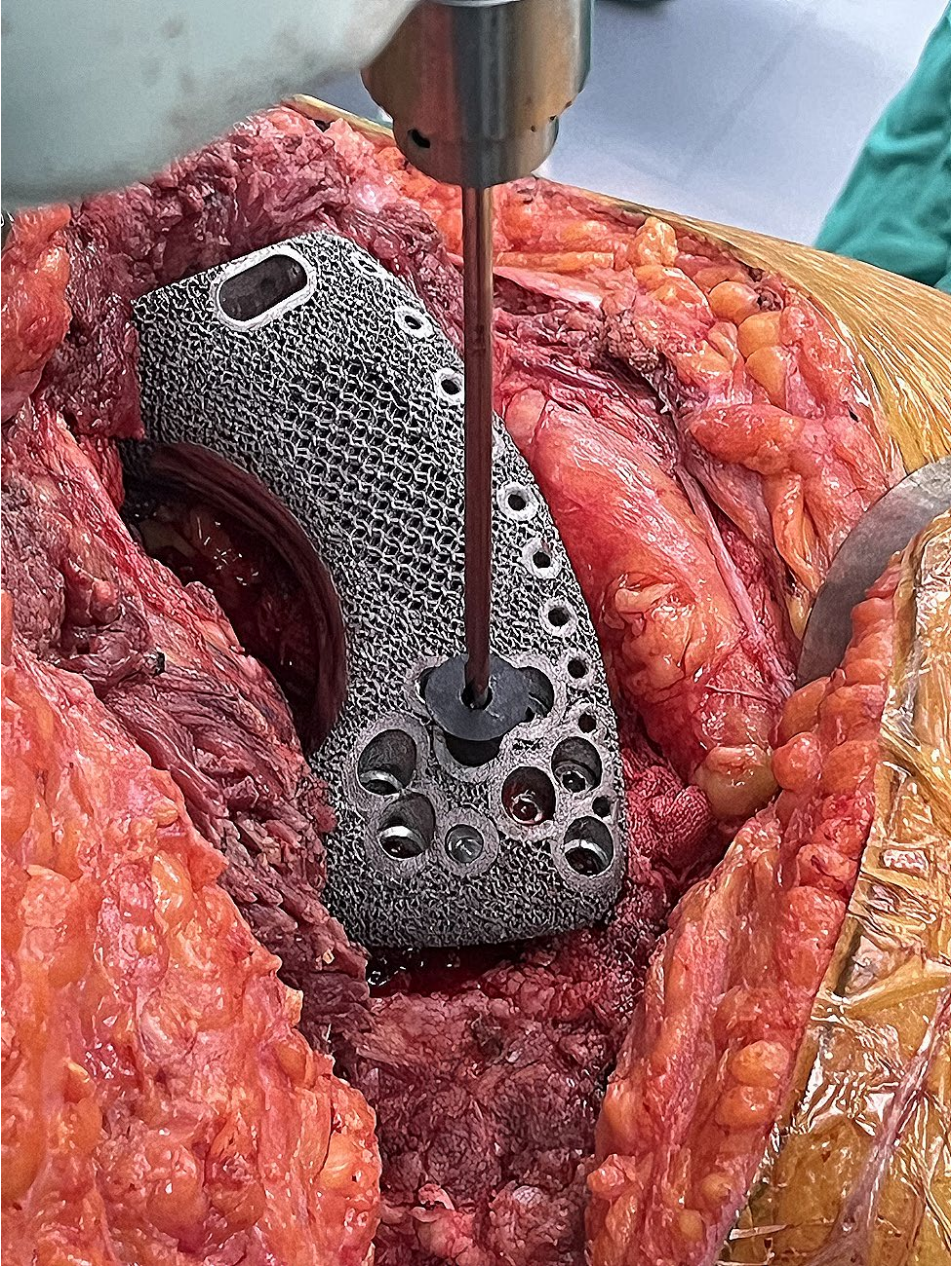
Intraoperative photo of using a drilling guide to improve the accuracy of screw insertion

All the surgeries were performed by the same senior surgeon (CQ T). Patients adopted a lateral or floating position, and an ilioinguinal incision was selected to expose the lesion and a pubic incision was added if necessary. After visualization of the surgical site, osteotomies were performed with the assistance of patient-specific bone-cutting guides. Then, the nylon trial prosthesis was used to check the resection accuracy. After satisfactory implantation of the prosthesis trial mold, the sacroiliac articular cartilage was removed using bone rongeurs, exposing the sacral trabecula. The true hemipelvis prosthesis was carefully inserted into the prepared bone defect. To improve the accuracy of screw insertion, a drilling guide was used to drill bony screw paths (Fig. 7). Then, screws were inserted to secure the prosthesis in place. Then, the constrained acetabular pad was fixed in the acetabular cup of the prosthesis, and the proximal femur prosthesis was implanted. The remaining muscles were sutured to the corresponding parts of the prosthesis through suture holes. Finally, the incision was sutured layer by layer, and a drainage tube for continuous negative pressure drainage was placed.

### Follow-up and outcomes assessment

A third-generation cephalosporin combined with vancomycin was given for ten to 14 days, followed by oral antibiotics for four weeks. The patient was followed monthly during the first 3 months, and every 3 months thereafter. Patient outcomes were assessed through a combination of clinical and radiological evaluations. Radiographic imaging, such as X-rays or CT scans, was used to monitor implant place, stability, and alignment. To evaluate osseointegration, tomosynthesis-Shimadzu metal artifact reduction technology (T-SMART) was utilized. Functional outcomes were assessed by the Musculoskeletal Tumor Society (MSTS) 93 score.

## Results

### Lattice hemipelvis prosthesis design

This new type of lattice hemipelvis prosthesis incorporated by three parts: an irregular porous part with the biomimetic property to facilitate osteointegration, a regular large porous part for reducing weight, and a solid load-bearing part. In total, 12 prostheses were created based on individual bone defects (Table 2). Through porous analysis in 3-Matic, multiple pore sizes were observed in the irregular porous lattice part (Figs. 4 and 5) with a wide distribution range (approximately 300–900 μm). The porosity analysis results showed that the average porosity of irregular porous structures was around 75.02% in the 12 prostheses, ranging from 74.7 to 75.6%.

### Clinical and radiologic outcomes

**Fig. 8:**
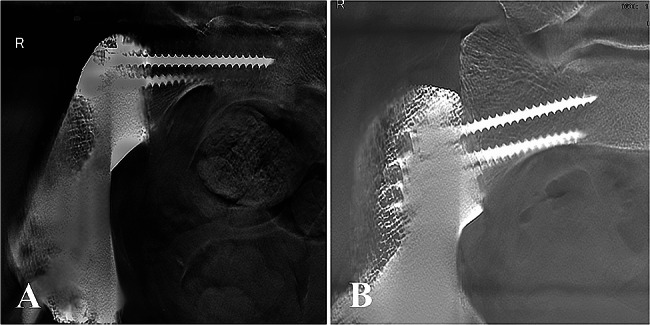
Osseointegration assessment according to T-SMART images of two patients, respectively

The average follow-up of 34.7 months, ranging 26 from to 43 months. No patient was lost to follow-up. One patient with Ewing sarcoma died of pulmonary metastasis 33 months after surgery while others were alive at the last follow-up. Postoperative radiographs showed that the prosthesis’s position was consistent with the preoperative planning. T-SMART images showed that the host bone was in close and tight contact with the prosthesis with no gaps at the interface, as shown in Fig. 8. Additionally, favorable bone density around the prosthesis was observed, without bone resorption or osteolysis. The average MSTS score was 21 at the last follow-up, ranging from 18 to 24. During the follow-up, three out of twelve patients experienced complications. Delayed wound healing seems to be the most frequent complication, which occurred in two patients. Both patients were cured after wound debridement. Dislocation occurred in one patient, requiring a closed reduction. In addition, there was no other complication requiring revision surgery or removal of the 3D-printed hemipelvis prosthesis, such as infection, screw breakage, and prosthesis loosening.

## Discussion

3D printing technology has the potential to revolutionize the field of pelvic reconstruction, allowing design freedom of the prosthesis for individual bone defects and fabrication of complex porous structures [2]. In the present study, we biomimetic designed a new 3D-printed lattice hemipelvis prosthesis with irregular porous structures inspired by the natural bone trabecular network. The preliminary results revealed multiple pore sizes with a wide distribution range in prostheses and subsequent good osteointegration in patients.

The prosthetic replacement has become a promising pelvic reconstruction treatment following tumor resection due to its flexible features, acceptable functional outcomes, early weight-bearing, as well as the possibility of rapid recovery [17]. Many studies focused on surgical technique and prosthesis design in periacetabular defect reconstruction and reported reasonable clinical and function outcomes [28–32]. However, for tumor resection without ilium preservation, fixing the prosthesis to the sacrum is surgical difficult because the flat surface of the sacroiliac joint is vulnerable to shear loading [11]. Despite the utilization of multiple screw fixation could achieve primary stability, long-term non-integration between the implant and host bone may result in a high incidence of reconstruction failure [17]. Under this situation, osseointegration is essential for the long-term stability of the hemipelvis prosthesis.

The porous structure acts as an osteoconductive scaffold for bone ingrowth [33] and therefore often suggested on the hemipelvis prosthesis to facilitate osseointegration and long-term stabilization [14]. It is noted that the porous structure design plays a significant role in osseointegration. Previous studies have reported that porous structures with pore sizes of 300 to 800 μm and high porosity (75%) are the most effective in promoting bone ingrowth [34–37]. In addition, a large number of studies have shown that the optimal pore size for improving osseointegration is determined by a specific range rather than a single value [38]. Therefore, irregular porous structures with different pore sizes are specially developed, and the property of facilitating osteointegration has been demonstrated in many vitro experiments [26, 27]. However, current hemipelvis prosthesis porous structures were often designed with regular lattice structures, which were composed of a single-element superposition and had a fixed pore size and porosity (Table 3). In the present study, to create the irregular lattice structure, dodecahedral units were set as randomized filling. Pore analysis results showed that multiple pore sizes with a wide distribution range (approximately 300–900 μm) in the irregular porous part (Figs. 4 and 5). In theory, this irregular porous structure had the advantage of promoting osteointegration compared to regular porous structures, which was significant for long-term stabilization. In our cases, osseointegration assessments according to T-SMART images indicated a high degree of bone-to-implant contact, along with favorable bone density around the prosthesis. During the follow-up period, good prosthesis stability was achieved without screw breakage or prosthesis loosening.

**Table 3:** Review of previous studies on the porous structure design of 3D-printed hemipelvis prosthesis

Study	Regular or Irregular	Lattice cell	Pore size	Porosity
Current	Irregular	Dodecahedron	300–900	75
Wang et al. ^[31]^	Regular	Dodecahedron	600	70
Peng et al. ^[19]^	Regular	NA	400	75
Wong et al. ^[32]^	Regular	NA	720	70
Han et al. ^[41]^	Regular	NA	400	60

NA: not available

In the present study, 3D-printed lattice hemipelvis prosthetic replacement following tumor resection allowed patients to restore favorable functional outcomes. The average MSTS score was 21 (range, 18–24) at the last follow-up, comparable with recent studies regarding using 3D-printed personalized hemipelvis prosthesis for pelvic reconstruction [2, 18, 20, 21, 39]. 3D-printed personalized prostheses were designed according to the mirror model of the contralateral hemipelvis. This procedure enabled the prosthesis to match individual bone defects completely while keeping a reasonable rotation center. In the present study, minimizing the ilium wing attributed to a much smaller prosthesis volume and made it easier for flap coverage. Additionally, the resected muscles around the hip were carefully repaired with suture holes on the prosthesis wing. Delayed wound healing was the most common complication in the present case series, occurring in two patients. In addition, dislocation occurred in one patient although muscles around the hip were reconstructed. In this study, no infection was detected during the follow-up. Besides strict aseptic procedures and rational use of antibiotics, osteotomies assisted with patient-specific bone-cutting guides shortened surgical time, which was believed to reduce the risk of infection. Additionally, the remaining muscles were sutured carefully to the corresponding parts of the prosthesis through suture holes. Reducing dead space around the prosthesis was another crucial strategy for preventing infection. It is noteworthy that the majority of previous studies have reported high rates of complications following similar surgeries [2, 40]. In light of the low incidence of complications observed in our study, it is imperative to acknowledge the limitations inherent in our sample size and follow-up duration. While our results are promising, further confirmation through longer follow-up periods and broader patient cohorts is warranted.

## Conclusion

In conclusion, the newly designed 3D-printed lattice hemipelvis prosthesis created multiple pore sizes with a wide distribution range and resulted in good osteointegration and favorable limb function.

### Electronic supplementary material

Below is the link to the electronic supplementary material.

Supplementary Material 1

## Data Availability

No datasets were generated or analysed during the current study.
