# Supplementary figures and images for: Biomimetic design and clinical application of Ti-6Al-4V lattice hemipelvis prosthesis for pelvic reconstruction
Source: J Orthop Surg Res. 2024 Apr 1;19:210. doi: 10.1186/s13018-024-04672-5 (PMC10983619; doi:10.1186/s13018-024-04672-5)

**Supplementary 1.** The formula for calculating the porosity of lattice hemipelvis prosthesis.


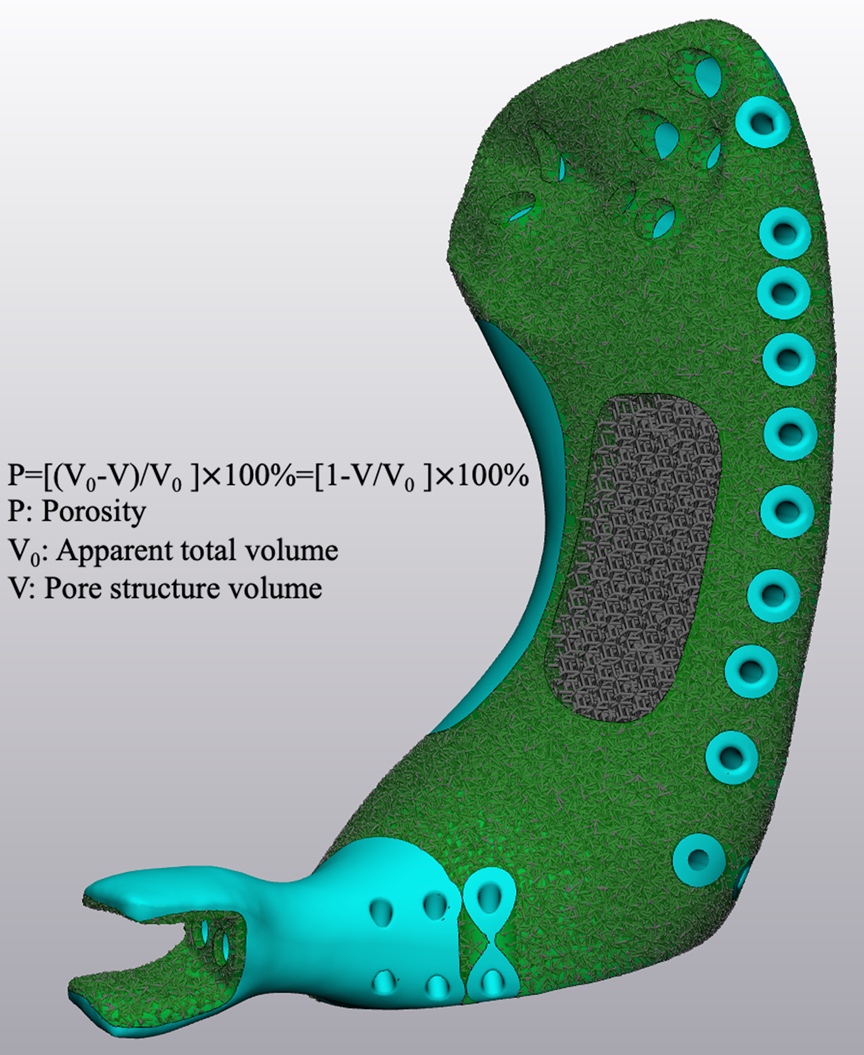

Supplement: Supplementary file 1 — Supplementary Material 1 [file 13018_2024_4672_MOESM1_ESM.docx]
